# Severity Assessment of Lower Respiratory Tract Infection in Malawi: Derivation of a Novel Index (SWAT-Bp) Which Outperforms CRB-65

**DOI:** 10.1371/journal.pone.0082178

**Published:** 2013-12-06

**Authors:** Edmund Birkhamshaw, Catriona J. Waitt, Michael Innes, Peter I. Waitt

**Affiliations:** 1 Birmingham College of Medicine, Birmingham University, Birmingham, United Kingdom; 2 Department of Molecular and Clinical Pharmacology, University of Liverpool, Liverpool, United Kingdom; 3 Department of Primary Care, Birmingham University, Birmingham, United Kingdom; 4 Department of Medicine, College of Medicine, University of Malawi, Blantyre, Malawi; D'or Institute of Research and Education, Brazil

## Abstract

**Objective:**

To assess the validity of CRB-65 (Confusion, Respiratory rate >30 breaths/min, BP<90/60 mmHg, age >65 years) as a pneumonia severity index in a Malawian hospital population, and determine whether an alternative score has greater accuracy in this setting.

**Design:**

Forty three variables were prospectively recorded during the first 48 hours of admission in all patients admitted to Queen Elizabeth Central Hospital, Malawi, for management of lower respiratory tract infection over a two month period (N = 240). Calculation of sensitivity and specificity for CRB-65 in predicting mortality was followed by multivariate modeling to create a score with superior performance in this population.

**Results:**

Median age 37, HIV prevalence 79.9%, overall mortality 18.3%. CRB-65 predicted mortality poorly, indicated by the area under the ROC curve of 0.649. Independent predictors of death were: Male sex, “**S**” (AOR 2.6); Wasting, “**W**” (AOR 6.6); non-ambulatory, “**A**” (AOR 2.5); Temp >38°C or <35°C, “**T**” (AOR 3.2); BP<100/60, “**Bp**” (AOR 3.7). Combining these factors to form a severity index (**SWAT-Bp**) predicted mortality with high sensitivity and specificity (AUC: 0.867). Mortality for scores 0–5 was 0%, 3.3%, 7.4%, 29.2%, 61.5% and 87.5% respectively. A score ≥3 was 84% sensitive and 77% specific for mortality prediction, with a negative predictive value of 95.8%.

**Conclusion:**

CRB-65 performs poorly in this population. The **SWAT-Bp** score can accurately stratify patients; ≤2 indicates non-severe infection (mortality 4.4%) and ≥3 severe illness (mortality 45%).

## Introduction

Lower respiratory tract infection (LRTI) is the third greatest cause of death globally, with 3.16 million deaths worldwide in 2008; half of these occurred in lower or middle income countries [1]. Amongst patients diagnosed with community acquired pneumonia (CAP) in sub-Saharan Africa, mortality rates of up to 23% have been reported [2]–[3]. Bacterial pneumonia in human immunodeficiency virus (HIV) positive patients is a World Health Organisation (WHO) stage 3 diagnosis, and is both a common index presentation and cause of death [3].

It has been recognised that clinical evaluation of patients with suspected CAP is prone to error in stratifying mortality risk [4]. To address this, two major clinical scoring systems were derived. The CURB-65 score considers five clinical variables (presence or absence of confusion, serum urea >7 mmol/l, respiratory rate >30 breaths/minute, blood pressure (BP) <90 mmHg systolic or <60 mmHg diastolic and age >65 years) and has become widely used in the UK [5]; CRB-65, a simplified version not requiring access to a laboratory, has similar validity, particularly in settings where expected overall mortality is low [6]–[7]. The Pneumonia Severity Index (PSI), most widely used in the USA, is a more complex scale relying on collation of 20 clinical, laboratory and radiologic variables [8]. A systematic review and meta-analysis by Loke and colleagues included 23 high quality validation studies of these scales, encompassing more than 22 thousand patients. Most were performed in well-resourced settings, and overall mortality was 7.6%. They concluded that PSI was the most sensitive test to exclude low risk patients, but required abundant resources and good infrastructure; CURB-65, or the simplified CRB-65 were also found to be robust clinical scales [9]. Chalmers and colleagues reported similar findings from a meta-analysis conducted according to the epidemiologically-focused MOOSE guidelines, but noted that ‘if the population of patients to which the score is being applied is significantly different from the original derivation it may be necessary to perform local recalibration of the score’ [10]. Importantly, this meta-analysis found the more simple CRB-65 to be equally accurate in mortality prediction when compared with PSI and CURB-65 [10]. However, it is notable that few studies have been conducted in low-resource settings, in environments where the mortality from CAP is high, or in communities where HIV rates are high. Furthermore, both CRB-65 and PSI were derived and validated using the endpoint of 30-day mortality [5], [7]–[8], whereas it is recognised that a significant proportion of patients in less developed countries may die within the initial 48 hours of admission [3].

We therefore aimed to determine the validity of the CRB-65 index in patients presenting to a Malawian hospital with a clinical diagnosis of CAP, and to identify variables predictive of in-hospital mortality. A simple assessment tool, based on objective, easily measurable clinical parameters, was developed, aiming to enable stratification of patients on initial presentation to hospital and to guide acute management.

## Methods

### Study Site and Patient Selection

The study was conducted on the medical admission ward of Queen Elizabeth Central Hospital (QECH), Blantyre, Malawi. Malawi is among the ten poorest countries worldwide in terms of both GDP and per capita income, and has approximately one doctor per 100 000 population [11]. A significant proportion of medical care is provided by clinical officers who have completed a three year training programme and who have limited opportunities for continued professional development.

All patients presenting with a clinical diagnosis of acute LRTI over a two month period in February and March 2010 were eligible. A presumptive diagnosis of CAP was made if; at least two recognised signs or symptoms of pneumonia were present [12]; with an illness duration of 21 days or less; and LRTI/CAP was the primary diagnosis recorded by the attending clinician All patients received empirical antibiotic treatment, based on local microbiological data collected by the Malawi-Liverpool-Wellcome Clinical Research Programme [13]–[14]. Patients were excluded from the study if LRTI was not the primary diagnosis; if they had recently been hospitalized within 14 days presentation; or if the patient had known malignancy.

Whilst international definitions of CAP require the presence of a new, presumed infective infiltrate on chest X-ray (CXR) [5], such confirmation is often not possible in low resource settings due to infrastructural constraints. Even when available, a delay of 24–48 hours from admission to performance of CXR means that the patients at highest risk of early death from LRTI [6], [15]–[16] may not receive radiological confirmation of diagnosis; additionally, the inability to perform portable CXR excludes patients who are too unwell to be transferred to the radiology department. We therefore chose a pragmatic, clinical case definition to identify a truly representative sample of patients, aiming to develop a stratification algorithm ideal for use at initial point of contact.

### Data Collection

Clinical data were collected using a proforma (a full list of variables is available as online Appendix). For repeatedly measured variables, the most abnormal value reported during the first 48 hours of admission was recorded.

‘Wasting’ was a nominal (yes/no) variable, and was defined as an overall assessment of nutritional status by the responsible clinician, taking into account various factors such as BMI and MUAC. Many of the patients were too unwell to stand and have formal BMI calculations undertaken, a challenge that has been noted in other African studies conducted in the acute setting [17]–[18]. To increase objectivity, mid-upper arm circumference (MUAC) was measured in 30% of cases, with less than 19 cm being defined as ‘wasting’; among these cases, MUAC strongly correlated with the potentially more subjective clinical judgement (Fisher's exact test; p<0.0001). ‘Confusion’ was defined as disorientation in time, place or person. Non-ambulatory was defined as being unable to independently mobilise on admission.

When CXR was available, these were independently interpreted by PW and EB. Where disagreement was noted, a visiting UK based consultant radiologist was asked to provide a final verdict.

### Primary Outcome

The primary outcome was inpatient mortality; longer term follow-up in the community was beyond the scope of this study.

### Statistical Analysis

Sample size was chosen, based on the anticipated 20% inpatient mortality suggested by previous studies conducted among similar populations [3], [14].

Continuously distributed variables were re-categorised into binary form using threshold values specified in existing severity prediction scores and literature pertaining to CAP [4]–[5], [8], [16]. A full list of threshold values applied is detailed in the online appendix.

The univariate association between each of 43 predictor variables and in-hospital mortality was analysed using Chi-squared tests (Table 1). Multivariable logistic regression, using a forward stepwise method, enabled identification of five independent associations with mortality (Table 2), which were combined to produce a new six-point clinical severity score, named SWAT-Bp.

**Table 1:** Univariate association between clinical variables and mortality.

Clinical feature	N/total*	(%)	Died	(%)	OR	p value
**Male sex**	116	48.3	28	24.1	2.15	**0.025**
**Age>45**	78	32.5	6	7.7	0.28	**0.004**
**Age>55**	50	20.8	4	8	0.35	**0.045**
**HIV positive**	155/194	79.9	32	20.6	1.77	0.266
**Confusion**	12	5	5	41.7	3.46	**0.032**
**RR>30/min**	151	62.9	31	20.5	1.58	0.205
**BP<100/60 mmHg**	88	36.7	29	33	4.83	**<0.001**
**BP<90/60 mmHg**	57	23.8	21	36.8	4.35	**<0.001**
**HR>120/min**	97	40.4	22	22.7	1.72	0.103
**T>38°C (axillary)**	111	46.3	25	22.5	1.78	0.089
**T<35°C**	19	7.9	9	47.4	4.92	**0.001**
**T>38 or <35°C**	134	55.8	34	25.4	3.94	**<0.001**
**Oxygen saturation <94%**	70/115	60.9	15	21.4	1.85	0.237
**Current smoker**	26	10.8	8	30.8	2.2	0.083
**Wasting**	76	31.7	31	40.8	8	**<0.001**
**Non-Ambulatory**	76	31.7	31	40.8	9.33	**<0.001**
**X-ray performed**	147	61.3	18	12.2	0.34	**0.002**
**X-ray: bilateral**	54/147	36.7	8	14.8	1.41	0.508
**X-ray: multizonal**	86/147	58.5	12	14	1.46	0.501
**Concurrent TB**	35	14.6	9	25.7	1.69	0.222
**HAART (in HIV^+^ patients)^+^**	39/128	30.5	4	10.3	0.39	0.103
**CPT (in HIV^+^ patients)∧**	73/155	47.1	16	21.9	1.39	0.343
**CRB-65>1 (‘mod-severe’)**	54	22.5	16	29.6	2.38	**0.015**
**SWATBp score >2 (‘severe’)**	82	34.2	37	45.1	17.74	**<0.001**

* Unless stated otherwise, N = 240 patients; +Highly Active Anti–Retroviral Therapy; ^∧^Co-trimoxazole prophylaxis.

**Table 2:** Clinical variables predictive of mortality in multivariate analysis.

Variable	AOR	95% CI	p-value
**Male sex (S)**	2.64	1.07 – 6.51	0.035
**Wasting (W)**	6.61	2.61 – 16.7	<0.001
**Non-Ambulatory (A)**	2.46	1.26 – 4.82	0.008
**Temp<35 or >38°C (T)**	3.18	1.19 – 8.50	0.022
**BP<100/60 mmHg (Bp)**	3.72	1.51 – 9.13	0.004

For each patient, a CRB-65 score(0–4) was calculated enabling comparison of the test characteristics for mortality prediction between both this existing score and the novel SWAT-Bp score (Table 3). The area under the receiver operating characteristic (ROC) curves enabled comparison of the accuracy of both indices.

**Table 3:** CRB-65 and SWAT-Bp scores; mortality prediction and test characteristics.

Score	No. features	N	Died (%)	No. features	Sensitivity (%)	Specificity (%)	PPV (%)	NPV (%)
**CRB-65**	0	64	4 (6.3)	≥0	100	-	18.3	2
	1	122	24 (19.7)	≥1	90.1	30.6	22.7	93.8
	2	47	13 (27.7)	≥	36.4	80.6	29.6	84.9
	3	6	2 (33.3)	≥3	6.8	98	42.9	82.4
	4	1	1 (100)	4	2.3	100	100	8
**SWAT-Bp**	0	29	0 (0)	≥0	100	-	18.3	-
	1	61	2 (3.3)	≥1	100	14.8	20.9	100
	2	68	5 (7.4)	≥2	95.5	44.9	28	97.8
	3	48	14 (29.2)	≥3	84.1	77	45.1	95.8
	4	26	16 (61.5)	≥4	52.3	94.4	67.6	90.7
	5	8	7 (87.5)	5	1	99.5	87.5	86.2

Data were analysed using SPSS Version 17.0 for Windows.

### Ethics statement

Ethical approval was obtained from the College of Medicine Research Ethics Committee, Malawi and the Department of Population Sciences Ethics Committee, University of Birmingham, to collect data from hospital records and pre-collected patient notes, without individual patient consent. All information was anonymised at the point of contact with patients' notes.

## Results

Two hundred and forty patients (48.3% male) were enrolled. Five patients (2%) were lost to follow-up and excluded from analysis. Median age was 37 (range 16–85, IQR 29–48) years. One hundred and fifty five were HIV positive (79.9% of those in whom status was known); 46 patients (19.2% of the cohort) remained of unknown status at the time of discharge or death. The overall inpatient mortality was 18.3%; of these deaths, 27% occurred during the first 48 hours of admission, and 75% during the first week.

All patients were treated with empirical antimicrobial agents, based on local microbiological research [13]–[14]. These included third generation cephalosporins, most frequently ceftriaxone; benzylpenicillin in combination with choloramphenicol; and oral amoxicillin. Choice of antibiotic was not associated with outcome, although a trend towards lower mortality was noted amongst patients who received oral amoxicillin (OR 0.34, p = 0.182); this most likely reflects clinical judgement that these were low-risk patients.

### Associations with in-hospital mortality

Thirteen variables were found to be significantly associated with adverse outcome, as detailed in Table 1. Independent associations with in-hospital mortality were male sex (‘S’); wasting (‘W’); being non-ambulatory (‘A’), axillary temperature of less than 35°C or greater than 38°C (‘T’) and blood pressure of less than 100 mmHg systolic or 60 mmHg diastolic (‘Bp’) (Table 2).

### Accuracy of CRB-65 score in predicting mortality

Mortality risk increased with CRB-65 score, rising from 6.3% in patients with ‘mild’ illness (score of 0), to 42.9% in those classified as ‘severe’ (scores of 3 or 4) [5]. However, only seven patients were identified as having severe illness by CRB-65, and a mortality of 21.9% occurred in patients classified as ‘moderate’ (score of 1 or 2); a single adverse feature on CRB-65 was associated with a mortality risk of 19.7% in our cohort. The sensitivity of a CRB-65 score ≥2 was 36.4% with a specificity of 80.6% in predicting mortality; the insensitivity is further demonstrated by a negative predictive value (NPV) of 84.9% at this level.

### Accuracy of SWAT-Bp score in predicting mortality

The relationship between SWAT-Bp score and mortality risk is shown in Table 3. No patient with a score of 0 died, compared with an 87.5% mortality amongst patients with a score of 5. A relatively even spread of patients across the six scores, together with a stepwise increase in mortality allowed classification into three risk groups: a score of 0 or 1 carried a mortality risk of 2.2%; a score of 2 represented intermediate risk with a 7.4% mortality; and a score of ≥3 had a mortality of 45.1%, with sensitivity and specificity of 84.1% and 77% respectively. The high NPV of 95.8% at this level illustrates the accuracy of this score in identification of patients with mild illness.

### Comparison of CRB-65 and SWAT-Bp scoring systems

**Figure 1:**
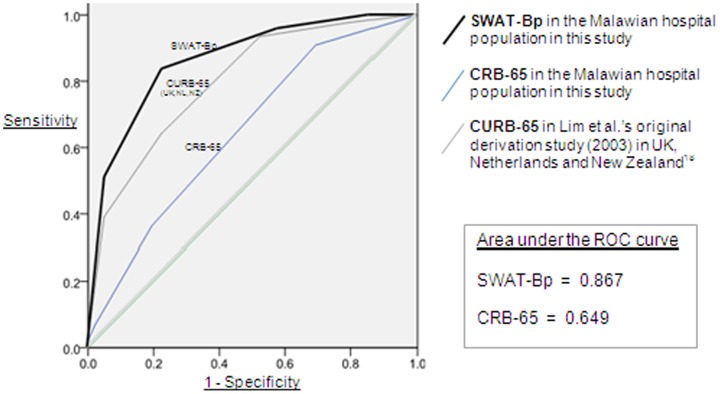
ROC curves for mortality prediction by SWAT-Bp and CRB-65 scores.

The ROC curves for CRB-65 (AUC 0.649) and SWAT-Bp (AUC 0.867) in this study population, and CURB-65 in the original derivation cohort of Lim and colleagues, illustrate the differences in accuracy in predicting mortality (Figure 1). The difference between the AUC for SWAT-Bp and CRB-65 in this population was highly statistically significant (P<0.001).

## Discussion

We present the derivation of a novel score, SWAT-Bp, which accurately predicts mortality risk in patients presenting to a Malawian hospital with a clinical presentation of LRTI and a presumed primary diagnosis of CAP. Patients with a score of 0 or 1 had a mortality risk of 2.2%, and could safely be triaged to receive outpatient treatment whereas a score of 2 represented intermediate risk (7.4% mortality) requiring admission for intravenous antibiotics and fluid therapy. Almost half of the patients scoring ≥3 according to SWAT-Bp died in hospital; identification of these patients at initial point of contact should allow the rational prioritisation of appropriate bundles of care aiming at early goal directed therapy [19].

This score identifies the adverse clinical features associated with mortality risk more accurately than the CRB-65 score which has been derived and validated amongst large cohorts of patients in industrialised nations [7], [9]–[10]. Meta-analysis of the existing predictive scores, CURB-65, CRB-65 and PSI has concluded with the caveat that these may require re-calibration in populations with significant differences from previously investigated cohorts [10], [20]. Despite this recommendation, few studies have been conducted in low-resource settings with high HIV seroprevalence.

The demographic differences in our population may partly explain the limitations of CRB-65. Only 10% of patients were aged over 65; in Malawi, the overall life expectancy is 53 years, a figure greatly influenced by the national HIV sero-prevalence of 11% [21]. Whilst confusion is a common complication of severe infections amongst elderly populations [22], it was a rare feature in our cohort, seen in only 12 (5%) patients. Investigating CAP in HIV positive Nigerians, a limitation of existing scores was found to be the skew towards reduced scores in young patients with relatively few known co-morbidities [23]; similarly, in Italy, PSI performed poorly in an HIV positive population, tending to generate low scores in patients who in fact had severe illness and high risk of death [24]. Studies from industrialised countries have shown the poor predictive performance of CRB65 and PSI in young patient cohorts overall [36], [37]. By contrast, simple indicators such as advanced wasting and the inability to walk unaided, which may be surrogates for advanced HIV infection in a young population, proved strong predictors of poor outcome in our study, findings paralleled by studies of adult medical admissions in Tanzania [17] and tuberculosis patients in Malawi [18].

In addition to being the first study seeking to identify a prognostic score among patients presenting with LRTI in this setting, a major strength of our study is the prospective and systematic collection of clinical information. The study was conducted in a real-life setting with several clinicians involved in patient care; many of the limitations encountered were reflective of the real-life environment in which we would propose our score to be used. The variables included in the model have the advantage of being easily measurable, objective physiological observations, which minimise risk of subjective bias. Furthermore, implementation of the score would be possible with minimal training and without any major resource implications for an already stretched healthcare system.

Although there is potential for subjectivity in the variable of ‘wasting’, we have demonstrated very close correlation between clinicians' assessments of wasting and the objective measure of MUAC (see Methods), thus illustrating the reproducibility of this variable in this setting. Whether this potential subjectivity impacts upon external validity will need to be investigated during the implementation stage of SWATBp.

The reliability of recording of physiological observations in this setting is a potential limitation of this study, in the context of its retrospective design. Many patients did not have initial variables recorded on first presentation. Therefore, to maximise the sensitivity of the scoring system, the most-abnormal value in the first 48 hours of admission was recorded. Future prospective studies will ensure variables are collected for all subjects on initial presentation, to validate the use of SWAT-Bp as an admission triage score.

It could be argued that the lack of radiological confirmation of diagnosis in 39% of our cohort undermines the significance of our findings. However, we sought to establish a pragmatic, clinical case definition similar to that seen in the emergency departments of hospitals across sub-Saharan Africa. Focusing our analysis on those patients who had CXR performed revealed a mortality of only 12% compared with 18.5% in the cohort as a whole; this likely reflects the reduced mortality risk in patients who were considered well enough to be transferred to the X-ray department, and who had survived for long enough for the investigation to be performed. Indeed, on univariate analysis, having had a CXR performed was associated with reduced mortality risk (OR 0.34, p = 0.002). Whilst we would not dispute the value of CXR in the diagnosis of CAP, there is increasing evidence that this investigation has limitations in populations with high rates of HIV. In a study of 49 HIV positive South Africans with a diagnosis of CAP, 85% of patients had radiological abnormalities on high resolution computed tomography (HRCT) that were not apparent on plain CXR [25]. Furthermore, recent consideration of the value of CXR in tuberculosis in Malawi has drawn attention to limitations in both technical quality and the interpretative skill of the majority of clinicians [26].

The difficulty in establishing clinical diagnoses with certainty has been highlighted in other Malawian studies [18], [27]. Even with significant research infrastructure and access to high quality microbiology laboratories, in patients presenting with an acute respiratory illness there will remain a large proportion in whom the final diagnosis is classified as ‘possible’ or ‘probable’ rather than ‘proven’ [14], [18], [27]. Up to a third of patients may have concurrent infection with more than one organism, and high rates of bacteraemia and mycobacteraemia have been reported [27]. Recent work has suggested that CURB-65 has utility as a generic risk score in adult acute medical admissions [28], to be used in a similar manner to the modified early warning score (MEWS) which has become integrated into routine management in the UK [29]. Hence, despite the limitations introduced by the limited diagnostic resources available to our study cohort, we would argue that the SWAT-Bp score has great merit in identifying high risk of death among patients with a clinical presentation which is consistent with pneumonia.

It is notable that physiological derangements noted in our high risk patients overlap with the features of Systemic Inflammatory Response Syndrome (SIRS). Studies conducted in the intensive care units (ICU) of industrialised nations have demonstrated a majority of patients who die from CAP to meet the clinical case definitions for severe sepsis [30], and that prognostic scores designed for both sepsis and pneumonia each have their merits and limitations in patients with pneumonia [31]. The initial management of severe sepsis was streamlined in 2008 by the Surviving Sepsis Campaign [32]; whilst these guidelines assume the availability of abundant resources to be delivered on an ICU setting, there exist valuable principles which can be extrapolated to the patient group we describe in this paper.

Being aware of the many challenges inherent in clinical medicine in low-resource settings, the WHO has developed protocols summarised as the Integrated Management of Adolescent and Adult Illness (IMAI) [33], which are analogous to the more well established Integrated Management of Childhood Illness (IMCI) pathways. These seek to enable less well trained clinicians with few resources to rapidly identify patients at high risk of poor outcome, to decide which patients require referral to a higher level of care, and provide guidance regarding the pragmatic empirical management of major medical conditions including CAP. Our score, based on a clinical diagnosis of CAP, would be a valuable addition to these pathways. Furthermore, given the overlap between LRTI, CAP, SIRS and sepsis, studies evaluating SWAT-Bp amongst patients presenting with a potential diagnosis of sepsis, regardless of its aetiology, would be informative.

A positive HIV status was not significantly associated with in-patient mortality, consistent with findings reported in a local cohort of TB patients [18]. HIV is without doubt a major risk factor for the acquisition of opportunistic infections such as CAP and TB, and the local impact of this is evidenced by 85% HIV seroprevalence among medical inpatients at QECH, the majority of whom have presented with an acute infectious illness (Waitt P, unpublished data). However, once bacterial infection is established, the processes of SIRS and sepsis carry high mortality irrespective of HIV status, as recently reported in Uganda [34]. That 19% of our patients did not have a known HIV diagnosis by the end of the study was not ideal, given the target for universal testing. However, this is rarely achievable in the acute setting due to the need for patient counselling and confidential testing in a clinical area where confidentiality can be maintained. An analysis of Nigerian patients diagnosed with CAP over a 5 year period revealed that only 35% of patients were tested for HIV [35]; similar limitations were acknowledged.

## Conclusion

We present a novel six-point prognostic score (SWAT-Bp) for prediction of mortality risk in patients presenting with a LRTI in a low-resource, high HIV seroprevalence population such as Malawi. This tool is ideal for stratifying the appropriate intensity of management of patients, leading to more effective use of resources and prioritisation of clinical care. Further validation is required among cohorts of similar patients, before consideration of more widespread use of the score.

## Supporting Information

Table S1 Full list of variables investigated and thresholds for abnormality.
(DOC)
