# Supplementary material for: Severity Assessment of Lower Respiratory Tract Infection in Malawi: Derivation of a Novel Index (SWAT-Bp) Which Outperforms CRB-65
Source: PLoS One. 2013 Dec 6;8(12):e82178. doi: 10.1371/journal.pone.0082178 (PMC3855704; doi:10.1371/journal.pone.0082178)
Supplement: Table S1 — Full list of variables investigated and thresholds for abnormality. (DOC) [file pone.0082178.s001.doc]

Supporting Information (Online Appendix)

Table S1: Full list of variables investigated and thresholds for abnormality

| **Variable** | **Threshold values for abnormality**  **(Informed by existing prognostic indices and current literature on CAP)^11,15,18,24,25,31^** |
| --- | --- |
| Sex | As reported in case notes for admission |
| Age | As reported in case notes for admission |
| HIV | As reported in case notes for admission |
| Confusion | Confusion recorded in case notes, defined as:  Disorientation in person / place / time |
| Resp. rate | > 30 / min and > 40 / min  (two separate variables entered into analysis) |
| Blood pressure | < 100 / 60 and < 90 / 60  (two separate variables entered into analysis) |
| Temperature | > 38^o^c or < 35^o^c (axillary) |
| Heart rate | > 120 / min |
| Oxygen saturation (%) | < 94 % on room air |
| Vital signs recorded in first 24 hours | BP, temp, HR, RR and O_2_ saturations recorded < 24 hours in case notes |
| Ambulatory | As reported in case notes for admission |
| Wasting  (MUAC) | As reported in case notes for admission  MUAC < 19cm defines severe wasting |
| Pleural effusion | As reported in case notes for admission |
| Bilateral signs | As reported in case notes for admission |
| Smoking status | As reported in case notes for admission; current / ex / never  (ex-smoker defined as abstinent for > 6 months) |
| Symptomatology on admission;  Duration | As reported in case notes for admission |
| Night sweats | As reported in case notes for admission |
| LOW | As reported in case notes for admission |
| Fever | As reported in case notes for admission |
| Pleuritic pain | As reported in case notes for admission |
| Haemoptysis | As reported in case notes for admission |
| Productive cough | As reported in case notes for admission |
| White cell count | < 4,000 or > 11,000 / mm^3^ |
| Urea | < 7 mmol / l |
| X-ray performed (features);  Consistent with pneu. |  |
|  | New opacification consistent with consolidation on chest  x-ray: assessed by at least two investigators |
| Bilateral changes | Assessed by at least two investigators |
| More than 1 zone | Assessed by at least two investigators |
| Comorbidities;  TB |  |
|  | As reported in case notes for admission |
| Chronic lung Disease | As reported in case notes for admission |
| Cardiovascular disease | As reported in case notes for admission |
| Diabetes Mellitus | As reported in case notes for admission |
| Dementia | As reported in case notes for admission |
| Chronic renal disease | As reported in case notes for admission |
| Chronic liver disease | As reported in case notes for admission |
| Rheu. Disorder | As reported in case notes for admission |
| Treatment during admission;  Antibiotic type | As reported in case notes for admission |
| IV/Oral route | As reported in case notes for admission |
| IV fluid support | As reported in case notes for admission |
| Co-trimoxazole prophylaxis  HAART | As reported in case notes for admission  As reported in case notes for admission |
